# An Overview of the Emergence of Plant Pathogen ‘*Candidatus* Liberibacter solanacearum’ in Europe

**DOI:** 10.3390/microorganisms11071699

**Published:** 2023-06-29

**Authors:** Vojislav Trkulja, Andrija Tomić, Slavica Matić, Nenad Trkulja, Renata Iličić, Tatjana Popović Milovanović

**Affiliations:** 1 Agricultural Institute of Republic of Srpska, Knjaza Miloša 17, 78000 Banja Luka, Bosnia and Herzegovina; vtrkulja@blic.net; 2 Faculty of Agriculture, University of East Sarajevo, Vuka Karadžića 30, 71123 East Sarajevo, Bosnia and Herzegovina; tomic_andrija@yahoo.com; 3 Institute for Sustainable Plant Protection, National Research Council, 10135 Turin, Italy; slavica.matic@ipsp.cnr.it; 4 Institute for Plant Protection and Environment, Teodora Drajzera 9, 11040 Belgrade, Serbia; trkulja_nenad@yahoo.com; 5 Faculty of Agriculture, University of Novi Sad, Trg Dositeja Obradovića 8, 21000 Novi Sad, Serbia; renatailicic@gmail.com

**Keywords:** zebra chip, yellowing, reddening, proliferation

## Abstract

In this paper, a comprehensive overview of the ‘*Candidatus* Liberibacter solanacearum’ presence in Europe was provided. The analyzed findings revealed that, since the first appearance of this pathogen in Finland and Spain in 2008, it has spread to 13 new European countries. Therefore, ‘*Ca*. L. solanacearum’ has spread very quickly across the European continent, as evident from the emergence of new host plants within the *Apiaceae*, *Urticaceae*, and *Polygonaceae* families, as well as new haplotypes of this pathogen. Thus far, 5 of the 15 ‘*Ca*. L. solanacearum’ haplotypes determined across the globe have been confirmed in Europe (haplotypes C, D, E, U, and H). Fully competent ‘*Ca*. L. solanacearum’ vectors include *Bactericera cockerelli*, *Trioza apicalis*, and *B. trigonica*; however, only *T. apicalis* and *B. trigonica* are presently established in Europe and are very important for plants from the *Apiaceae* family in particular. Moreover, psyllid species such as *B. tremblayi*, *T. urticae*, and *T. anthrisci* have also been confirmed positive for ‘*Ca*. L. solanacearum’. Constant monitoring of its spread in the field (in both symptomatic and asymptomatic plants), use of sensitive molecular diagnostic techniques, and application of timely management strategies are, therefore, of utmost importance for the control of this destructive pathogen.

## 1. Introduction

‘*Candidatus* Liberibacter solanacearum’ Liefting et al. (syn. ‘*Candidatus* Liberibacter psyllaurous’ Hansen et al.) is a phloem-limited, Gram-negative, filamentous, and unculturable bacterium that belongs to the order Rhizobiales, the class Alphaproteobacteria, and the phylum Proteobacteria. The complete ‘*Ca*. L. solanacearum’ genome is 1.3 Mb in size and is taxonomically related to ‘*Candidatus* Liberibacter asiaticus’, a suspected causal agent of the citrus huanglongbing [1]. Although ‘*Ca*. L. solanacearum’ is primarily spread by psyllid insect vectors [2,3,4], it can also be disseminated through infected vegetative material, such as potato tubers [5]. This polyphagous bacterium is an economically important pathogen for various host plants in the Americas, New Zealand, and Europe [6]. 

‘*Ca*. L. solanacearum’ causes a potato disease known as “zebra chip” that was first described in 1994 in Mexico, where it caused significant economic damage [7,8,9,10]. The bacterium was also recorded in 2000 and 2001 on potato crops in Latin America [7] and then in Texas and Nebraska in the US in 2003 [11], where it caused considerable yield losses. Initially, the pathogen associated with “zebra chip” was identified as the phytoplasma ‘*Candidatus* Phytoplasma americanum’ [12]. The true cause of the disease remained unknown until 2008, when two research groups, from New Zealand and the US, respectively, started working on establishing the exact etiology of the disease independently from one another. The research group from New Zealand, led by Liefting et al. [8,13], first discovered the bacterium in tomato and pepper, and then in potato, as well as in several other plant species of the *Solanaceae* family. Consequently, they named the new species ‘*Candidatus* Liberibacter solanacearum’ in reference to the plant host family from which it was isolated. The research team from the US, led by Hansen et al. [2], discovered this pathogen in tomato plants and the psyllid *Bactericera cockerelli* (Schulz) and named the bacterium ‘*Candidatus* Liberibacter psyllaurous’ in recognition of its association with psyllid yellows [4]. By comparing the 16S rRNA gene sequences, it was subsequently determined that the American isolates of ‘*Ca*. Liberibacter psyllaurous’ represented the same species as the bacterium found in New Zealand denoted ‘*Ca*. Liberibacter solanacearum’ [14,15,16,17].

To date, the presence of ‘*Ca*. L. solanacearum’ has been reported in the western and central United States (Texas, Nebraska, Colorado, Kansas, Wyoming, New Mexico, Arizona, Nevada, California, Idaho, Oregon, and Washington) [2,3,4,9,14,18], Mexico [19,20], Central America (Guatemala, Honduras, Nicaragua, and El Salvador) [3,4,21,22], and New Zealand [8,13,15], where the zebra chip disease renders potato tubers practically unusable, and thus causes economic losses equivalent to millions of US dollars [13,23,24,25]. The disease manifests as dark flecking throughout the flesh of affected tubers, which is attributed to vascular tissue necrosis. Occasionally, severe streaking of the medullary ray tissues occurs and becomes more pronounced in tubers after frying, giving rise to its characteristic name, “zebra chip”. The appearance of these symptoms makes the diseased tubers unsuitable for both fresh sale as well as processing. Foliar symptoms in plants affected by zebra chip vary considerably but include an upward rolling of the basal portion of young leaves, chlorosis, purple top, aerial tubers, leaf scorch, zigzag-shaped stems, axillary bud proliferation, swollen nodes, shortening of internode length, vascular discoloration, plant wilting, and premature aging [5,9,26]. 

**Figure 1:**
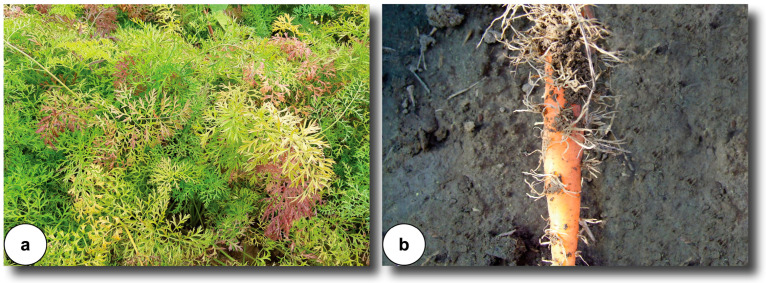
‘*Candidatus* Liberibacter solanacearum’: (**a**) symptoms on carrot leaves (foto T. Popović Milovanović); (**b**) symptoms on carrot root (foto R. Iličić).

‘*Ca*. L. solanacearum’ is a species that causes great damage to plants from the *Solanaceae* family (potato and tomato in particular) across the globe but also affects plants from the *Apiaceae* family in Europe (Figure 1). For this reason, in the EPPO region, in 2012, ‘*Ca*. L. solanacearum’ was placed on the A1 list as the pathogen recommended for regulation as a quarantine pest [27]. According to Harrison et al. [25], ‘*Ca*. L. solanacearum’ is responsible for severe damage to the crops from the *Solanaceae* family in North America and New Zealand and is a causative agent of “zebra chip” in potato. This Gram-negative bacterium inhabits the phloem tissue of host plants, which leads to disruption of its function, resulting in the appearance of disease symptoms. 

‘*Ca*. L. solanacearum’ currently represents one of the greatest phytosanitary threats to the production of numerous plant species in Europe, primarily those from the *Apiaceae* family. After the first identification of this bacterium in carrot crops in Finland and Spain in 2008, the bacterium spread very quickly in other countries and infected new host species, causing significant economic damage.

## 2. Distribution of ‘*Ca*. L. solanacearum’ in Europe

**Figure 2:**
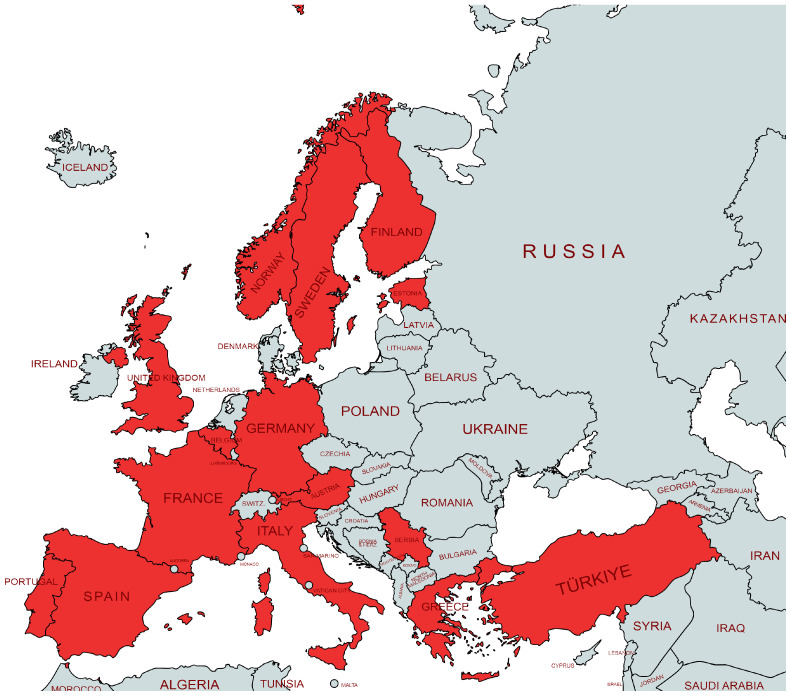
‘*Candidatus* Liberibacter solanacearum’: distribution map in Europe; 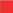 present.

In Europe, ‘*Ca*. L. solanacearum’ was first described in 2008 in Finland [28], followed by Spain [29]. Subsequently, the presence of this bacterium was established in Sweden [30] and Norway [31] in 2011; France in 2012 [32]; Germany in 2014 [33]; Greece, Italy, and the UK in 2016 [34,35]; Austria in 2019 [36]; Belgium [37,38], Estonia [39], and Portugal [40] in 2017; and Serbia [41] and Turkey [42] in 2020 (Figure 2).

The first record of ‘*Ca*. L. solanacearum’ presence was made in Finland in August 2008, whereby the affected carrot plants (*Daucus carota* L.) were also infected by the carrot psyllid *Trioza apicalis* Förster (Hemiptera: Triozidae). The affected plants exhibited leaf curling, along with yellow and purple discoloration, root and shoot growth retardation, and secondary root proliferation. This was also the first evidence that this bacterium could infect plants that do not belong to the *Solanaceae* family [28,43]. In the years that followed, the bacterium was detected in the vector *T. apicalis* in various regions of Finland, where it also infected carrot plants and caused significant yield reductions [44,45,46].

In the same year, but also during 2009 and 2010, the first record of ‘*Ca*. L. solanacearum’ in Spain was made for carrot plants grown in Tenerife (Canary Islands), as well as in central Spain [29,47,48]. 

In Sweden, this bacterium was first identified in carrot plants in 2011 [30,49,50]. Although 70% of investigated carrot plots in the southern parts of Sweden showed ‘*Ca*. L. solanacearum’ symptoms, at the time, the disease was declared as contained within a limited area [50]. 

In Norway, the presence of this bacterium was also confirmed in 2011 in symptomatic and asymptomatic carrot plants grown in the southeastern parts of the country [31,51]. Soon, new field surveys and molecular analyses showed that the bacterium had spread and had become domesticated throughout different carrot production regions (Østfold, Akershus, Vestfold, Oppland, and Hedmark) in Norway [52,53]. The symptoms the diseased plants exhibited included leaf discoloration and curling, which was present in 10–100% of the affected plants [52].

In France, ‘*Ca*. L. solanacearum’ presence was confirmed in 2012 in carrot plants [32,54] and manifested as leaf yellowing, stunting, and proliferation of dwarfed shoots with bushy tops and a dense hairy growth on secondary roots, whereby such symptoms were present in 50–90% of examined crops. Immediately after this discovery, certain phytosanitary measures were taken to prevent the further spread of this pathogen, including a ban on the commercialization of seeds produced in infected fields, destruction and plowing of plant residues, and disinfection of all seed harvesting machinery [54]. However, these measures were proven ineffective, as analyses of plant samples belonging to the *Apiaceae* family, such as carrot, celery, chervil, fennel, parsnip, and parsley collected during the 2012–2016 period confirmed the presence of ‘*Ca*. L. solanacearum’ in different regions of the country, thus confirming the polyphagy of this bacterium [55].

In Germany, the pathogen was first recorded in 2014 in diseased carrot plants in the Lower Saxony–Niedersachsen region [33,56], which exhibited the same symptoms as those identified in carrot plants in Finland [28,33]. In 2017, the bacterium was also discovered for the first time in the *Trioza urticae* vector in Germany [57].

In Austria, ‘*Ca*. L. solanacearum’ presence was first established in 2015 in carrot and celery plants [58], followed by hogweed (*Heracleum sphondylium* L.) plants in 2019 [36]. In 2017, symptoms were also detected in parsley and parsnip [36]. 

In Greece, the bacterium was first confirmed in carrot root hairs in 2016 [34,59], while in the same year, the presence of this pathogen was confirmed in Italy in carrot seeds and a year later in carrot plants in Sicily [35,60,61].

In the UK, the bacterium was first detected in 2016 in Scotland, where it was confirmed in the seeds of three parsley varieties—Comun 3, Moss Curled 2, and Plain leaved [46,62]. During the 2015–2016 growing season, the bacterium was also confirmed in the collected *Trioza anthrisci* Burckhardt vector specimens, marking the first detection of this pathogen in a vector in this country [63]. Its presence was subsequently confirmed in asymptomatic carrot plants grown in the field [64].

‘*Ca*. L. solanacearum’ was detected in Belgium for the first time in 2017 in carrots grown in the field as well as seeds of the Nerja variety [37,38]. A month later, the bacterium was confirmed for the first time in Estonia in the vector *Trioza apicalis* [39], and in 2019 and 2020, it was also identified in the vector *T. urticae* [65]. Also in 2017, this bacterium was confirmed for the first time in a commercial carrot field in the municipality of Olho Marinho in Portugal [40].

In Turkey, ‘*Ca*. L. solanacearum’ was first confirmed in 2020 in carrot and parsley plants grown in the Central Anatolia region [42,66]. 

In Serbia, the bacterium was confirmed for the first time in 2020 in Begeč, a town located in the southern part of the Bačka region, where it affected carrot plants. This was the first finding of this pathogen in the former Yugoslavia territory [41,67].

Based on their research on the presence of ‘*Ca*. L. solanacearum’ in seed batches from the SASA and Warwick collections of *Apiaceae* species from Europe, but also from other parts of the world, Monger and Jeffries [68] concluded that this bacterium had been present in Europe much earlier than previously indicated. By analyzing carrot seeds of the Lobbericher variety from France and Germany dating back to 1973, the authors confirmed that ‘*Ca*. L. solanacearum’ had been present in carrot seeds in Europe for at least 50 years. Findings yielded by the examination of SASA collections further revealed that 17 of the 23 tested carrot varieties from Europe were positive for ‘*Ca*. L. solanacearum’, while in another collection, 9 of the 17 carrot varieties from Europe were also positive for this bacterium. The positive varieties originated from Denmark, England, France, Germany, the Netherlands, Great Britain, and the Czech Republic. In addition to cultivated carrots, wild carrot seeds from *D. carota* and *D. aureus* varieties (from Lebanon), as well as seeds of European celery, parsley, and parsnip varieties, gave positive results for the presence of ‘*Ca*. L. solanacearum’, indicating that this pathogen had been present in the seeds of species from the *Apiaceae* family since ancient times but reached an economically significant level once environmental conditions were favorable for the vector population to increase to a sufficient size to cause epidemics such as those that had occurred in Finland and Spain.

The seeds of celery varieties that were positive for ‘*Ca*. L. solanacearum’ originated from the Netherlands, Italy, Germany, and the Netherlands, while one variety was either from Italy or Slovenia. Infected parsley was from Great Britain, Denmark, and Germany, and parsnips originated from the Czech Republic, England, Italy, and the Netherlands. Although the authors tested small quantities of seeds, most varieties from European countries—carrot, parsley, and parsnip in particular—were positive for the presence of ‘*Ca*. L. solanacearum’. Even though Monger and Jeffries [68] reported positive results for Denmark, the Netherlands, and the Czech Republic, the presence of this pathogen on plants, their seeds, or in vectors was not substantiated by any independent reports from these countries.

Due to the increased phytosanitary risk that would arise from the introduction and further spread of ‘*Ca*. L. solanacearum’ in the territory of Slovenia, Lithuania, and the Netherlands, special surveillance programs have been implemented for this quarantine phytopathogenic bacterium, but its presence in these three European countries has not been determined to date [27,69]. 

As can be surmised from the preceding discussion, since its first appearance in 2008, ‘*Ca*. L. solanacearum’ has been found in 15 European countries.

## 3. Hosts of ‘*Ca*. L. solanacearum’ in Europe

‘*Ca*. L. solanacearum’ has been found on a large number of host plants across the globe, particularly those from the *Solanaceae* family, including potato (*S. tuberosum*), tomato (*S. lycopersicum*), eggplant (*Solanum melongena* L.), pepper (*Capsicum* sp.), chili (*C. frutescens* L.), tamarillo (*S. betaceum* Cav.), Jerusalem cherry (*S. pseudocapsicum* L.), tobacco (*Nicotina tabacum* L.), cape gooseberry (*Physalis peruviana*), and goji berry (*Lycium barbarum* L.), as well as some weeds such as bittersweet nightshade (*Solanum dulcamara* L.), European black nightshade (*S. nigrum* L.), silverleaf nightshade (*S. elaeagnifolium* Cav.), black nightshade (*S. ptychanthum* Dun.), and thorn-apple (*Datura stramonium* L.), which also belong to the family after which this bacterium was named [3,13,15,19,20,21,22,27,45,70,71,72,73,74,75]. Laboratory research conducted in the US indicates that this bacterium can be successfully transferred to new weed species from the *Solanaceae* and *Convolvulaceae* families, confirming the importance of understanding its host spectrum in the prevention of its spread. In particular, it is important to identify weed species that act as its hosts because they can serve as pathogen and vector reservoirs even in the absence of cultivated host plants, and thus provide a source of inoculum for new infections [76]. 

However, throughout Europe, the most important hosts on which this bacterium was first established and on which it regularly emerges are plants from the *Apiaceae* family, such as carrot (*Daucus carota* L.), celery (*Apium graveolens* L.), parsnip (*Pastinaca sativa* L.), and parsley (*Petroselinum crispum* (Mill.) Fuss) [3,29,30,32,36,41,42,44,47,52,55,77], as shown in Table 1. As previously noted, Monger and Jeffries [68] have determined the presence of ‘*Ca*. L. solanacearum’ in the seed collections of various European carrot, celery, parsley, and parsnip varieties, as well as in two wild carrot varieties from Lebanon (*D. carota* and *D. aureus*). As indicated in Table 1, in addition to the *Apiaceae* family, this pathogen also infects plants belonging to the *Urticaceae* and *Polygonaceae* families [78,79,80]. It is also worth noting that this bacterium is successfully transferred from one vegetation to another through infected seeds, which thus represent the primary inoculum for the next growing season and allow infection to be spread to new areas. In 2015, Bertolini et al. [81] provided one of the first reports of successful ‘*Ca*. L. solanacearum’ transmission through carrot seeds on European soil. Based on their experiments carried out with infected seeds in France, the authors further stated that the pathogen could be transmitted under experimental conditions through dodder (*Cuscuta campestris* Yunck.), periwinkle (*Catharanthus roseus* (L.) G. Don) (Table 1) and other herbaceous plants. However, these findings are countered by the results reported by Loiseau et al. [82], who also experimented with infected carrot seeds but failed to establish transmission of pathogens from seeds to seedlings (plants) during the study period. Their results were subsequently corroborated by Mawassi et al. [83].

In Spain, plants from the *Apiaceae* family, primarily carrot and celery, are the main ‘*Ca*. L. solanacearum’ hosts (Table 1), which is a highly relevant finding, given that these crops can be grown throughout the year [77]. Infected carrots typically exhibited leaf curling, along with yellow, bronze, and purple leaf discoloration, shoot and tap root stunting, and secondary root proliferation [29,47]. Teresani et al. [74] found that, during 2009 and 2010, celery grown in the Alicante region was heavily infected with ‘*Ca*. L. solanacearum’, which was attributed to higher temperatures during the summer period. Symptoms not previously observed on the celery cultivars ‘Loretta’, ‘Monterrey’, and ‘Imperial’ celeriac of the *Apium graveolens* var. *dulce* (Mill.) variety manifested in the form of an abnormally large number of shoots, as well as stem twisting and yellowing, which was the first finding of a new host for ‘*Ca*. L. solanacearum’. On the other hand, the symptoms of the ‘Brillant’ variety of turnip-rooted celery *A. graveolens* var. *rapaceum* (Mill.) were in the form of stunted growth. Apart from carrot and celery, the bacterium was also detected by Alfaro-Fernández et al. [84] in parsley and parsnip, as shown in Table 1. According to these authors, in parsnip, disease symptoms included leaf yellowing and proliferation, as well as secondary root stunting and proliferation with early root senescence, whereas parsley exhibited yellowing, along with reddening and proliferation of leaves. Based on their investigation involving a large number of weeds growing along the perimeter of carrot and celery fields in Spain, Alfaro-Fernández et al. [77] established the limited presence of this bacterium, suggesting that it does not frequently infect weeds. According to EPPO [85], haplotype E of ‘*Ca*. L. solanacearum’, which predominantly infects plants from the *Apiaceae* family, was also detected in potato tubers in storage facilities in Spain.

In France, since its first detection in carrot plants, the bacterium has spread and has been identified on other hosts from the *Apiaceae* family (Table 1). For example, Hajri et al. [55] confirmed that, in the 2012–2016 period, in addition to carrot and celery as its main hosts, ‘*Ca*. L. solanacearum’ was confirmed in four other plant species from this family, namely parsley, parsnip, and fennel (*Foeniculum vulgare* Mill.), as well as in the weed variety chervil (*Anthriscus cerefolium* (L.) Hoffm.) (Table 1). These findings indicate that this bacterium has a wider range of hosts within the *Apiaceae* family than was previously assumed. Infected carrot specimens exhibited leaf yellowing, accompanied by stunting and proliferation of dwarfed shoots with bushy tops and a dense hairy growth on secondary roots. On other crops such as celery, parsley, and parsnip, similar symptoms were also recorded, including proliferation of shoots, yellowing and curling of leaves, proliferation of secondary roots, and stunted plant growth, while weed plants did not exhibit the characteristic disease symptoms [32,55]. 

In Finland, as a part of their research conducted in 2011, 2012, and 2013, Haapalainen et al. [45] found that cultivated and wild potatoes that grew next to or in a carrot crop field in which ‘*Ca*. L. solanacearum’ presence was confirmed were also infected by this bacterium, despite the absence of the characteristic “zebra chip” symptoms. The authors further noted that a significantly lower concentration of bacteria was found on potatoes without symptoms compared to infected carrots that exhibited leaf discoloration. In another study, Haapalainen et al. [78] confirmed ‘*Ca*. L. solanacearum’ presence on nettle (*Urtica dioica* L.) as a new host, indicating that it can be found in a range of hosts from the *Urticaceae* family [80]. Moreover, as shown in Table 1, cow parsley (*Anthriscus sylvestris* (L.) Hoffm.)—a weed species and the main host of the vector *Trioza anthrisci*—was positive for ‘*Ca*. L. solanacearum’, suggesting that this plant can be a suitable reservoir of this pathogen along the edges of cultivated plots. In cow parsley and parsnip samples, the pathogen was detected in the roots and leaf petioles, indicating their systemic infection [78]. In addition to these hosts, ‘*Ca*. L. solanacearum’ was also established on weed species such as wild buckwheat (*Fallopia convolvulus* (L.) Á. Löve) and pale persicaria (*Persicaria lapathifolia* (L.) Gray) from the *Polygonaceae* family (Table 1) growing in carrot and parsnip plots, which was the first record of these plants as ‘*Ca*. L. solanacearum’ hosts [79].

In Austria, during the 2014–2017 period, the disease was found in carrot and celery plants, while in 2018, ‘*Ca*. L. solanacearum’ was detected only in carrots. In 2017, its presence was confirmed in parsley and parsnip, and in 2019 in hogweed (*Heracleum sphondylium*) as a new host [36] (Table 1).

In other European countries where the presence of ‘*Ca*. L. solanacearum’ was confirmed, carrot was the dominant host, while in Turkey, this pathogen was also found in parsley (Table 1). Given that the bacterium is polyphagous and that it globally infects plants from other families (of both cultivated and weed variety), it is necessary to establish continuous constant supervision and monitoring of the potential spectrum of new hosts that can serve as reservoirs for the pathogen’s survival and sources for subsequent infection.

## 4. Haplotypes of ‘*Ca*. L. solanacearum’ in Europe

Thus far, the existence of 15 ‘*Ca*. L. solanacearum’ haplotypes that differ in geographic distribution and host plants, but are closely related to vector feeding and range, has been confirmed [86]. Haplotypes are identified on the basis of sequence analysis of single nucleotide polymorphisms (SNPs) within genes on chromosome 16S rRNA, 16S-26S ISR-intergenic spacer region, 16S/23S ISR, and 50S rplJ and rplL ribosomal protein genes [84,87], as well as by examining multilocus sequence typing markers (MLST) and simple sequence repeats (SSRs) [88]. Accordingly, the 15 ‘*Ca*. L. solanacearum’ haplotypes are denoted as A, B, C, D, E, F, G, H, H(Con), U, Cras1, Cras2, Aph1, Aph2, and Aph3 [28,33,70,74,78,79,86,87,88,89,90,91].

‘*Ca*. L. solanacearum’ haplotypes A and B are associated with the diseases they cause on plants from the *Solanaceae* family (e.g., *S. tuberosum*, *S. lycopersicum*, *Capsicum annuum*, and *Nicotiana* spp.), as well as with the vector *B. cockerelli*, which transmits the disease [26,81,84,87,92,93]. These two haplotypes are very important for the American continent and New Zealand but have not yet been confirmed in Europe (Table 2). Nelson et al. [87] were the first to report on the presence of haplotype A in Central America and New Zealand, after which haplotypes A and B were confirmed in the USA and Mexico [81,84,94]. These haplotypes cause zebra chip symptoms in potato, resulting in leaf wilting, chlorosis, and curling, as well as discoloration and premature death in the above-ground parts, while delayed development, wilting, and chlorosis are its most prominent characteristics in tomato [95]. 

‘*Ca*. L. solanacearum’ haplotypes C, D, and E are associated with diseases in plants from the *Apiaceae* family [55,78,79,93]. Specifically, haplotype C has been reported to infect carrot crops in European countries (such as Austria, Finland, Norway, Sweden, the UK, Germany, and Estonia), while haplotypes D and E have been reported in several southern European countries (Greece, Italy, Portugal, and Spain), as well as in Belgium, France, and the UK [26,40,64] (Table 2). Haplotype C was found in a carrot crop in Finland and was associated with *T. apicalis*, due to which carrot yield was reduced by up to 100% [23,28,44,87,92,96]. Haplotype C was also found in potato plants growing on the perimeter of carrot fields, which did not exhibit any zebra chip symptoms [96]. In Finland, haplotype C was detected in parsley, whereby affected plants exhibited mild leaf discoloration symptoms [78]. This haplotype was also determined in a single European black nightshade (*Solanum nigrum*) specimen in southwestern Finland [78]. However, haplotype C has also been confirmed in Sweden, Norway, Austria, Germany, and the UK, where it is associated with the vectors *B. trigonica* and *T. apicalis* [30,31,33,36,39,63,92,93,97] (Table 2). By analyzing the seeds stored in SASA and Warwick collections, Monger and Jeffries [68] determined its presence in celery seeds of the Alba GS variety collected in 1997 in Germany, as well as in parsley seeds of the Bravour and Curlina varieties obtained from the UK between 1990 and 2005.

Haplotype D was identified for the first time in a carrot crop in the Canary Islands (Spain) and was associated with the vector *B. trigonica* [47,92,97]. Subsequently, the same haplotype was found in the carrot crops grown in France [32,81], Greece [34,59], and Belgium [38], as well as in carrot seeds in Italy [35] and Finland [78] (Table 2). After Bertolini et al. [81] reported that haplotypes D and E were found in carrot seeds and plants in France, Hajri et al. [55] determined their presence in carrot, celery, parsley, and chervil (*A. cerefolium*) also grown in France. These plants exhibited symptoms of shoot proliferation, yellowing and curling of leaves, secondary root proliferation, and stunted growth, while these typical symptoms were absent in parsnip and fennel (*F. vulgare*) that tested positive for ‘*Ca*. L. solanacearum’. 

Similar symptoms of carrot plants infected with these strains were recorded by Othmen et al. [98]. In Scotland, haplotypes D and E were first identified in parsley seeds [46,62]. Moreover, by analyzing the seed collection of a large number of carrot varieties originating from all over the world dating from 1973 to 2006, Monger and Jeffries [68] established the presence of haplotype D in 14 carrot varieties, whereby ten of these samples were collected from six European countries (Germany, France, England, Netherlands, Italy, and Czechoslovakia) between 1973 and 1999. The same authors also determined the presence of haplotype D in the collections of celery seeds of Di Verona varieties from 1980 originating from Italy and Slovenia, as well as Dorato d’Asti from 1994 and Sigfrido from 1998 originating from Italy, parsley varieties Korte from 2002 originating from Germany, and the parsnip variety Kamo from 2013, originally from the Czech Republic.

The first report of haplotype E relates to celery grown in Spain, which was soon followed by its identification in carrot and potato plants [85,99]. Similar to haplotype D, this haplotype is associated with the B. trigonica vector [74], as confirmed by [100], by conducting experiments with carrot and celery plants. According to Antolínez et al. [101], haplotype E transmission in carrot and potato can also be carried out by the psyllid B. nigricornis, indicating that further research is needed in Europe to establish the frequency and significance of transmission in field conditions. In France, the presence of haplotype E was first determined in carrot seeds and plants [81], after which it was confirmed in celery, parsley, and chervil plants [55] and in commercial carrot crops grown in Portugal [40]. In Italy, haplotype E was confirmed in the seeds of the commercial carrot varieties Berlicum and Mezza Lunga Nantese [35], while in Scotland, it affected the seeds of the parsley varieties Moss Curlet 2 and Plain Leaved [46,62]. Meanwhile, investigating the presence of ‘*Ca*. L. solanacearum’ in the seed collection of a large number of carrot, celery, parsley, and parsnip varieties, Monger and Jeffries [68] confirmed the presence of this haplotype in parsley seeds of the Commun 2 variety from 2005 originating from England, and Bravour from 2007 originating from Denmark, as well as in the parsnip variety Dlouhy Bily from 2008, originally from the Czech Republic.

‘*Ca*. L. solanacearum’ haplotype U was named after nettle plants (*Urtica dioica*, member of the *Urticaceae* family), in which it was first identified in Finland [78]. As these plants usually grow as weeds along the edges of carrot crop plots, this was the first haplotype that was found to infect plants that do not belong to the *Solanaceae* or *Apiaceae* family (Table 2). At the same time, this haplotype was confirmed in the insect *Trioza urticae,* frequently found on nettle [78,80]. Haplotype H is thought to have been confirmed in the same vector in Germany [57]. While haplotype U was also confirmed in the UK in *T. uticae* and *U. dioica* [64], no evidence of its presence in other hosts presently exists.

‘*Ca*. L. solanacearum’ haplotypes F and G were discovered next and have since not been confirmed in Europe. Haplotype F was found by Swisher Grimm and Garczynski [89] in Southern Oregon (US) in potato tubers that exhibited zebra chip symptoms, including streaking and dark medullary rays. These authors posited that, like haplotypes A and B, haplotype F can be pathogenic for all plants from the *Solanaceae* family. Shortly thereafter, haplotype G was discovered in Southern California (US) in herbarium specimens of the perennial native plant Solanum umbelliferum Eschsch. which belongs to the *Solanaceae* family and is also the natural host of the vector *B. cockerelli* [102]. According to Mauck et al. [102], this haplotype has been present in the US since at least 1970 (Table 2).

‘*Ca*. L. solanacearum’ haplotypes H and H(Con) are the two currently described H haplotypes due to their simultaneous publication, although these two H haplotypes are genetically distinct and have different psyllid and plant hosts [86].

Haplotype H was first identified in Finland in 2018 in diseased carrot and parsnip plants [79] (Table 2). The infected carrot plants exhibited symptoms similar to those caused by haplotype C (strong change in the leaf color), while in parsnip, slightly reddish petioles and slight wrinkling of the leaves were accompanied by the appearance of brown color at root tips in some plants. This haplotype was also confirmed in weeds that grow in carrot crops such as wild buckwheat and pale persicaria (manifesting as reddish rim on the leaves), which are members of the *Polygonaceae* family, as confirmed for the first time by [79]. However, at present, information on the competent vectors of this haplotype is limited.

The H(Con) haplotype was discovered in the US before the H haplotype as a part of the investigation conducted by Torres et al. [103] involving field bindweed (*Convolvulus arvensis* L.) and sweet potato (*Ipomoea batatas* (L.) Lam), both of which are members of the *Convolvulaceae* family, due to which it was later named H(Con). Although these authors determined the presence of ‘*Ca*. L. solanacearum’ in these two host plants, they did not describe the established bacterium as a new haplotype. This was later completed by Contreras-Rendón et al. [90], who identified a novel Convolvulaceae-associated ‘*Ca*. L. solanacearum’ haplotype in both host plants and named it haplotype H based on the DNA polymorphism analysis of two 16S rRNA gene sequences originating from the US, which were deposited in GenBank by Torres et al. [103].

To distinguish this haplotype from the previously identified haplotype H, Sumner-Kalkun et al. [86] proposed denoting it as haplotype H(Con), as this nomenclature was chosen by Contreras-Rendón et al. [90] based on further analysis of 16S ribosomal gene regions. However, as the full set of ribosomal genes necessary for the assignment of new haplotypes has not been sequenced, further research is needed for the full acceptance of this haplotype. Thus far, haplotype H(Con) has not been confirmed in Europe (Table 2).

‘*Ca*. L. solanacearum’ haplotypes Cras1 and Cras2 were first detected in Scotland (UK) (Table 2) in the insects *Craspedolepta nebulosa* Zetterstedt and *Craspedolepta subpunctata* Förster, which belong to the *Aphalaridae* family [86]. These haplotypes were named in reference to the genus to which these insects belong [64,86]. Within the Cras1 haplotype, two variants (Cras1a and Cras1b) were distinguished, which differ from other Cras1 sequences by 3 SNPs in the 50 s rplJ/rplL gene region. Haplotype Cras1a was found in *C. nebulosa* and *C. subpunctata*, whereas Cras1b was identified in *C. subpunctata* [86].

Haplotypes Aph1, Aph2, and Aph3 are the most recently identified ‘*Ca*. L. solanacearum’ haplotypes [91] and were found in the US in collected species of psyllids of the genus Aphalara, after which they were named. The presence of haplotype Aph1 was confirmed in the psyllid species *Aphalara loca* Caldwell, while haplotype Aph2 (its two variants, Aph2a and Aph2b) was identified in *A. persicaria*, and the presence of all three haplotypes was confirmed in *A. curta* Caldwell. Haplotype Aph2 was divided into two subhaplotypes—Aph2a and Aph2b—based on the differences in the nucleic acid sequences for the 16S rRNA gene [91]. To date, none of these haplotypes has been confirmed in Europe (Table 2).

## 5. Vectors of ‘*Ca*. L. solanacearum’ in Europe

‘*Ca*. L. solanacearum’ is a bacterium that is transmitted to plants from the *Solanaceae* and *Apiaceae* families by insects that belong to psyllids (aphids), which feed on plant sap from the phloem. Psyllids enable bacteria to be transmitted in the field from one plant to another, as well as to new hosts and to new geographical areas. Although psyllids (Hemiptera, Psylloidea) also feed on plant sap from xylem, phloem sap is extremely important for the completion of their lifecycle on some plants [104,105,106]. 

Psyllids are small insects, 1–5 mm in length, that feed on phloem sap. They have worldwide distribution and reproduce mainly on perennial dicotyledonous plants, in which lifecycle lasts up to a month depending on the environmental factors [57,100,107]. Their lifecycle begins with the mating of individuals of different sexes, after which the female lays 100–1000 eggs on host plants. Adults can fly within a 1 km range, which can be extended under favorable wind conditions [100]. The lifecycle duration, number of eggs, flight range, number of generations per year, and the hosts on which they overwinter depend on the psyllid species and environmental conditions [26]. Their host range is not particularly large and is limited to a few closely related plant species or genera, due to which they are considered highly host-specific [107,108].

In the field, ‘*Ca*. L. solanacearum’ primarily spreads from one plant to another through a vector. The transmission of this bacterium is similar to that of persistent-propagative plant viruses, as it requires that the bacterium persists in the vector tissues for a certain period, allowing it to multiply before being transmitted to a new plant [81,109,110,111]. More precisely, the insect must swallow the pathogen, which must pass through the alimentary canal wall and move through the hemolymph or other tissues to the salivary glands before it can be transferred with the salivary secretions to a new host plant during feeding, which is characteristic of other ‘*Candidatus* Liberibacter’ species [26,112].

According to Antolínez et al. [113], only insects that reach the phloem vessels and continuously feed on plant sap from the phloem can effectively transmit this phloem-limited bacterium. The results reported by Sandanayak et al. [114], Munyaneza et al. [115], and Teresani et al. [100] further indicate that, for successful ‘*Ca*. L. solanacearum’ transmission, psyllid needs to capture the bacterium with a stylet from the phloem, and to successfully introduce it into new tissue or inoculate a new plant, it needs to introduce the bacterium into the phloem elements of the sensitive host plant through saliva. The success and efficiency of transmission depend primarily on the vector feeding behavior and the spectrum of hosts on which the vector feeds [116]. The longer the salivation (secretion of saliva) period before phloem feeding, the higher the probability of ‘*Ca*. L. solanacearum’ transfer [100,116]. The previously mentioned authors concur with this observation, as in their studies, higher transmission rates were recorded when the vector lingered for a longer period, salivated, and then fed on the phloem. Alvarado et al. [117] further stated that the intensity of physiological responses in potato plants is correlated with the ‘*Ca*. L. solanacearum’ levels present in psyllids.

The fully competent ‘*Ca*. L. solanacearum’ vector species that have been established to date include *Bactericera cockerelli*, *Trioza apicalis*, and *B. trigonica*, whereby the latter two are established and very important in Europe, especially for plants from the *Apiaceae* family.

*Bactericera cockerelli* (Hemiptera: Triozidae), also known as potato flea, is the most important vector for ‘*Ca*. L. solanacearum’ transmission to plants from the *Solanaceae* family [118,119,120]. The ability of *B. cockerelli* to transmit this pathogen to healthy host plants was first established by Hansen et al. [2]. Findings reported by Munyaneza et al. [115] indicate that the potato flea is capable of successfully transferring the bacterium to carrot plants even though they are not its natural host, highlighting its potential danger. *B. cockerelli* is a vector of particular importance for the American continent and New Zealand [2,3,16,25,76,121], as it has not been established in Europe thus far [26,78].

*Trioza apicalis* (Hemiptera: Triozidae), also known as carrot flea, is the main vector for ‘*Ca*. L. solanacearum’ transmission in Finland and other northern and central European countries, where carrots are grown intensively and where the carrot psyllid populations are extensive [28,30,52,78]. In these countries, *T. apicalis* is a dangerous pest, as it causes great economic damage to carrot crops, while manifesting in the form of leaf curling and yellow and purple discoloration, stunted shoot and root growth, and secondary root proliferation [23,28,96]. Munyaneza et al. [23,52] were the first to establish a potential connection between *T. apicalis* and ‘*Ca*. L. solanacearum’ based on molecular analyses, which revealed the presence of this bacterium in insects collected in 2009 in the field in Finland, and in 2011–2012 in Norway. According to Nissinen et al. [122], although both male and female *T. apicalis* individuals successfully transmit ‘*Ca*. L. solanacearum’ to healthy carrot plants, females tend to cause more damage, which results in a significant carrot root weight and leaf mass reduction, accompanied by an increase in the number of curled leaves. Infected *T. apicalis* populations collected in the field were tested by Haapalainen et al. [45], who noted that these insects managed to successfully transmit the bacterium to carrot plants, while infected carrot fleas grown under controlled (greenhouse) conditions failed to transmit the bacterium, as successful vector transmission of the bacterium requires a high ‘*Ca*. L. solanacearum’ titer in the vector.

*Bactericera trigonica* (Hemiptera: Triozidae) is a psyllid that is a competent ‘*Ca*. L. solanacearum’ vector in the Mediterranean region of Spain (as well as in the Canary Islands) and France [32,47,54,74,92,113,116]. The presence of this vector on plants from the *Apiaceae* family was also determined in Serbia [123]. *B. trigonica* feeds mainly on plants from the *Apiaceae* family, especially carrot [124]. This insect is particularly important for plants from the *Apiaceae* family due to its high activity and relatively easy access to the phloem sap compared to plants from the *Solanaceae* family [100]. Teresani et al. [100] also detected ‘*Ca*. L. solanacearum’ in 20% of eggs laid by females carrying the bacterium. On the other hand, Antolínez et al. [113] found that, while in *T. apicalis* gender played a role in the pathogen transmission, this was not the case for *B. trigonica* where symptom development in inoculated plants was not affected by gender. In laboratory tests of ‘*Ca*. L. solanacearum’ transmission to carrot plants via *B. trigonica*, Keshet-Sitton et al. [125] showed that the inoculation period of 3–7 days is sufficient and that the greater the number of insects that contain the bacteria, the faster the symptoms develop and the more destructive they are. The same authors reported that inoculations of young carrot plants with 10 or 20 psyllids per plant resulted in faster symptom development compared to inoculations with only two psyllids per plant. Based on their findings, Mawassi et al. [83] emphasized the success of transmission with *B. trigonica*, as the post-inoculation period of 30–45 days at 24–28 °C (which is typical for carrot growth conditions) was sufficient for symptom emergence. Successful transfer of this bacterium to carrot and celery plants was confirmed by Antolínez et al. [116], who also noted its preference for those plants when compared to potatoes. When it comes to oviposition, the pest always chose plants of the *Apiaceae* family, while in potatoes, the oviposition was very low. Moreover, in the tests carried out by these authors, *B. trigonica* efficiently transferred the bacterium to potato plants, highlighting the risk of transmission from plants of the *Apiaceae* family to potatoes or other plants of the *Solanaceae* family.

*Trioza urticae* (Hemiptera: Triozidae) is a psyllid that was discovered for the first time to carry the bacterium ‘*Ca*. L. solanacearum’ in Europe in Finland during 2015–2016 by Haapalainen et al. [78]. In 2017, its presence was also detected in Germany, albeit only in captured *T. urticae* individuals that fed on nettle, which grew as a weed along the perimeter of carrot plots [57,80]. This finding indicates that fully competent ‘*Ca*. L. solanacearum’ vectors can be found within weeds belonging to the *Urticaceae* family, which may lead to the emergence of new hosts visited by the vectors, and hence to additional alternative hosts and pathogen reservoirs in nature [78].

*Trioza anthrisci* (Hemiptera: Triozidae) is a psyllid confirmed by Satakunta Haapalainen et al. [45] in 2015 in the Tavastia Proper and Satakunta regions in Finland to be positive for ‘*Ca*. L. solanacearum’. These authors, nonetheless, stated that, as *T. anthrisci* is a pest that is in Finland, usually present on *Anthriscus sylvestris*, it presently has no significance for the transmission of this pathogen to potato plants because it emerges in early spring in this country (with the first warm days of April), while the peak egg laying activity occurs at the end of May and the beginning of June when potatoes in Finland have not yet sprouted or are just sprouting [45]. *T. anthrisci* individuals that were positive for the presence of ‘*Ca*. L. solanacearum’ were also found in Sweden and Scotland in 2017 by Sjölund et al. [126] and EPPO [63], respectively. Their findings further indicated that, as adults of this species overwinter on conifers and evergreen shrubs, these aspects should be considered when developing measures aimed at combating this pest.

In the Mediterranean region of Spain, not only *B. trigonica*, but other species from this genus—such as *B. tremblayi* (Wagner) and *B. nigricornis* (Foerster)—were also positive for the presence of ‘*Ca*. L. solanacearum’ and should thus be recognized as the potential vectors of this bacterium [94,116]. According to the EFSA [26], *B. nigriornis* is present in Western Europe and Asia, while *B. tremblayi* has been found in Greece, Italy, Serbia, Switzerland, Turkey, and Iran. In research conducted by Antolínez et al. [116], *B. tremblayi* tested positive for this bacterium but failed to transmit it to carrot plants. Based on previous literature reports, *B. nigricornis* and *B. tremblayi* are not fully capable of performing the vector role for ‘*Ca*. L. solanacearum’ [94,116]. However, Antolínez et al. [101] recently found that *B. nigricornis* is capable of transmitting ‘*Ca*. L. solanacearum’ to carrot and potato, indicating that further research is needed to understand its transmission in field conditions in Europe. In addition to these pests, on the Spanish island of Tenerife (Canary Islands), other unidentified psyllid species (*Bactericera* sp.) were positive for ‘*Ca*. L. solanacearum’ [94].

## 6. Control of ‘*Ca*. L. solanacearum’ in Europe

In Europe, ‘*Ca*. L. solanacearum’ management strategies mostly rely on the phytosanitary measures such as prevention of the entry and spread of the bacterium and its vectors via potato, solanaceous seed, and plant import and export, along with the imposition of mandatory post-entry quarantine [26]. Moreover, in the case of an outbreak, a demarcated area should be established, comprising an infected area and a buffer zone (with the boundaries at least 2 km away from the infected zone). All marked zones should be treated with insecticides, appropriate herbicides should be applied to host debris and weeds, and tests for bacterium presence should be conducted after the harvest [127].

As indicated by Babu et al. [128] and Nissinen et al. [129], who adopted some components of integrated pest management (IPM)—such as chemical, natural, and biocontrol strategies aimed at psyllid vectors—monitoring and early-season detection of psyllids is essential in the pest management and spreading of ‘*Ca*. L. solanacearum’. For vector monitoring, Babu et al. [128] also advised placing yellow sticky belts, water traps, and neon-green traps around crops. More recently, Jerinić Prodanović et al. [123] also found that after the appearance of the first psyllid forms, treatments with appropriate synthetic insecticides can be effective, especially when accompanied by systemic insecticide application during the intensive plant growth phase. Since weed species serve as reservoirs for ‘*Ca*. L. solanacearum’, herbicide use is also recommended, along with good agricultural practices, such as removal of alternative hosts and on-farm biosecurity and hygiene measures aimed at preventing the introduction, establishment, and spreading of pests and diseases [130]. Based on the findings yielded by the POnTE Project [130], an integrated approach was developed, leveraging mechanical tools such as nets, insecticide products (both synthetic and ‘natural’ such as kaolin), and technologies such as drip irrigation. While any combination of the aforementioned strategies has been shown to be more effective than its individual components, Nissinen et al. [129] found that kaolin treatment can effectively reduce psyllid reproduction in terms of the number of eggs as well as nymphs. These authors also demonstrated that an insect-proof mesh effectively prevented the feeding by *B. trigonica* and thus hindered the ‘*Ca*. L. solanacearum’ transmission. In practice, integrated control programs comprising products that target different psyllid life stages (maltodextrin, natural pyrethrin, *Beauveria bassiana*, and acetamiprid) have been proven effective in ‘*Ca*. L. solanacearum’ control. However, scientific investigations are increasingly focusing on biological control strategies and natural products in order to eliminate the adverse impacts of insecticidal treatments on crops and prevent the emergence of resistant psyllids. 

In conclusion, the number of established host plants and ‘*Ca*. L. solanacearum’ haplotypes, as well as potential and new vectors in Europe and across the globe is continually being revised, as the appearance of the bacterium in new hosts and new areas is expected, given that the current climate changes create favorable conditions for the emergence, development, and spread of this pathogen in Europe.

## Figures and Tables

**Table 1:** List of host plants on which ‘*Candidatus* Liberibacter solanacearum’ has been confirmed in European countries.

Scientific Names of Plant	Family	FI	ES	SE	NO	FR	DE	GR	AT	IT	EE	BE	PT	RS	TR	UK
*Daucus carota* L. (carrot)	*Apiaceae*	+	+	+	+	+	+	+	+	+	+	+	+	+	+	+
*Apium graveolens* L. (celery)	*Apiaceae*	–	+	–	–	+	–	–	+	–	–	–	–	–	–	-
*Petroselinum crispum* (Mill.) Fuss (parsley)	*Apiaceae*	–	+	–	–	+	–	–	+	–	–	–	–	–	+	+
*Pastinaca sativa* L. (parsnip)	*Apiaceae*	+	+	–	–	+	–	–	+	–	–	–	–	–	–	–
*Foeniculum vulgare* Mill. (fennel)	*Apiaceae*	–	–	–	–	+	–	–	–	–	–	–	–	–	–	–
*Anthriscus cerefolium* (L.) Hoffm. (chervil)	*Apiaceae*	–	–	–	–	+	–	–	–	–	–	–	–	–	–	–
*Heracleum sphondylium* L. (hogweed, cow parsnip)	*Apiaceae*	–	–	–	–	–	–	–	+	–	–	–	–	–	–	–
*Anthriscus sylvestris* (L.) Hoffm. (keck, cow parsley)	*Apiaceae*	+	–	–	–	–	–	–	–	–	–	–	–	–	–	–
*Solanum tuberosum* L. (potato)	*Solanaceae*	+	+	–	–	–	–	–	–	–	–	–	–	–	–	–
*Solanum nigrum* L. (black nightshade)	*Solanaceae*	+	–	–	–	–	–	–	–	–	–	–	–	–	–	–
*Urtica dioica* L. (nettle)	*Urticaceae*	+	–	–	–	–	–	–	–	–	–	–	–	–	–	–
*Fallopia convolvulus* (L.) Á. Löve (wild buckwheat)	*Polygonaceae*	+	–	–	–	–	–	–	–	–	–	–	–	–	–	–
*Persicaria lapathifolia* (L.) Gray (pale persicaria)	*Polygonaceae*	+	–	–	–	–	–	–	–	–	–	–	–	–	–	–
*Cuscuta campestris* Yunck. (dodder)	*Convolvulaceae*	–	–	–	–	+	–	–	–	–	–	–	–	–	–	–
*Catharanthus roseus* (L.) G. Don (periwinkle)	*Apocynaceae*	–	–	–	–	+	–	–	–	–	–	–	–	–	–	–

Notes: + present; – absent; FI—Finland; ES—Spain; SE—Sweden; NO—Norvey; FR—France; DE—Germany; GR—Greece; AT—Austria; IT—Italy; EE—Estonia; BE-Belgium; PT—Portugal; RS—Serbia; TR—Turkey; UK—United Kingdom. Source: Munyaneza et al. [30,31]; Loiseau et al. [32]; Bertolini et al. [81]; Ilardi et al. [35]; Alfaro-Fernández et al. [29,77,84]; Hajri et al. [55]; Holeva et al. [34]; Haapalainen et al. [45,78,79]; Lethmayer and Gottsberger, [36]; Trkulja et al. [41]; Karahan et al. [42]; EPPO [27,37,39,40,48,49,51,54,56,58,59,61,63,66,67,85].

**Table 2:** List of ‘*Candidatus* Liberibacter solanacearum’ haplotypes present in European countries.

Haplotypes	AT	FI	NO	SE	DE	BE	FR	IT	PT	ES	GR	UK
Haplotype A	–	–	–	–	–	–	–	–	–	–	–	–
Haplotype B	–	–	–	–	–	–	–	–	–	–	–	–
Haplotype C	+	+	+	+	+	–	–	–	–	–	–	+
Haplotype D	–	+	–	–	–	+	+	+	–	+	+	+
Haplotype E	–	–	–	–	–	–	+	+	+	+	–	+
Haplotype U	–	+	–	–	+	–	–	–	–	–	–	+
Haplotype F	–	–	–	–	–	–	–	–	–	–	–	–
Haplotype G	–	–	–	–	–	–	–	–	–	–	–	–
Haplotype H	–	+	–	–	–	–	–	–	–	–	–	–
Haplotype H(Con)	–	–	–	–	–	–	–	–	–	–	–	–
Haplotype Cras1	–	–	–	–	–	–	–	–	–	–	–	+
Haplotype Cras2	–	–	–	–	–	–	–	–	–	–	–	+
Haplotype Aph 1	–	–	–	–	–	–	–	–	–	–	–	–
Haplotype Aph 2	–	–	–	–	–	–	–	–	–	–	–	–
Haplotype Aph 3	–	–	–	–	–	–	–	–	–	–	–	–

Notes: + present; – absent; AT—Austria; FI—Finland; NO—Norway; SE—Sweden; DE—Germany; BE—Belgium; FR—France; IT—Italy; PT—Portugal; ES—Spain; GR—Greece; UK—United Kingdom. Source: Alfaro-Fernández et al. [29,84]; Teresani et al. [74]; Munyaneza et al. [33]; Monger and Jeffries [62]; Hajri et al. [55]; Holeva et al. [34]; Haapalainen et al. [78,79]; De Jonghe et al. [38]; EFSA [26]; EPPO, [27,40,59,64]; Grimm et al. [91].

## Data Availability

Not applicable.
